# Sociodemographic factors associated with major depressive episodes and suicidal ideation among emerging adults with diabetes in the U.S

**DOI:** 10.3389/fendo.2023.1276336

**Published:** 2023-12-07

**Authors:** Sandhya Yadav, Young-Rock Hong, Sarah Westen, Nicole M. Marlow, Michael J. Haller, Ashby F. Walker

**Affiliations:** ^1^ Department of Health Services Research Management, and Policy, University of Florida, Gainesville, FL, United States; ^2^ Department of Clinical and Health Psychology, University of Florida, Gainesville, FL, United States; ^3^ Department of Pediatrics, University of Florida, Gainesville, FL, United States

**Keywords:** diabetes, disparity, suicidal ideation, mental illness, major depressive episode, LGBTQ

## Abstract

**Background:**

Research focused on disparities related to mental health comorbidities, especially among emerging adults with diabetes, is limited. Identifying associated factors of disparities could inform policy decisions to make diabetes-related interdisciplinary care more accessible for vulnerable groups.

**Method:**

Using data from the National Survey on Drug Use and Health (2015-2019), we examined disparities in presence of major depressive episode (MDE) and suicidal ideation among emerging adults with diabetes. Survey design-adjusted bivariate and multivariable logistic regression models were used for statistical analyses.

**Results:**

The study included 1,125 emerging adults (18-25 years old), with a history of type 1 diabetes (T1D) or type 2 diabetes (T2D). After controlling for sociodemographic and health-related characteristics, we found lower odds of having past-year major MDE for non-Hispanic Black (AOR, 0.42, p=0.032) compared to their non-Hispanic White counterparts. Females were 3.02 times more likely to have past-year MDE than males (AOR, 3.02, p=0.004). The odds of having past-year MDE were 1.96 times higher among individuals who identified as LGB (lesbian, gay, bisexual) (AOR, 1.96, P=0.038). There were no statistically significant disparities in suicidal ideation related to race/ethnicity, sex, education, and family income. However, individuals who identified as LGB had significantly higher likelihood of suicidal ideation than their heterosexual counterparts (AOR, 2.47, P=0.004).

**Conclusion:**

Significant disparities related to MDE and suicidal ideation exist based on race/ethnicity, gender, and sexual orientation. Integration of a mental health professional into the multidisciplinary diabetes care team is critical for effective management of comorbid mental health conditions in younger patients with diabetes.

## Highlights

- Higher risk of Major Depressive Episodes (MDE) for women and sexual minority (lesbian, gay, bisexual (LGB)) emerging adults with diabetes.
- LGB emerging adults have an increased risk of suicidal ideation compared to their heterosexual counterparts.
- Among emerging adults, Non-Hispanic Blacks have lower odds of experiencing MDE compared to non-Hispanic Whites.
- Integration of mental health professionals into diabetes care team is crucial for managing comorbid mental health conditions in emerging adults with diabetes.

## Introduction

Diabetes is one of the fastest-growing health challenges of the 21^st^ century in the United States (US) and around the world. In 2020, 10.5% of the US population (34.2 million) was estimated to have diabetes (1). Incidence and prevalence of diabetes both type 1 diabetes (T1D) and type 2 diabetes (T2D) is increasing dramatically among adolescents and emerging adults (18-25 years) (2). Diabetes is a challenging condition to live with and mental illness often co-exists with diabetes (3, 4).

Emerging adults with diabetes are particularly at risk as they enter a critical developmental stage in life, often referred to as emerging adulthood (5), characterized by multiple life transitions—from high school to college/workforce, from living with parents to living by themselves, from already established social and peer support system to building new interpersonal relationships in college/workplace (5, 6). These challenges are further complicated by an abrupt change in their professional diabetes care as they enter adulthood, which involves a shift from their pediatric diabetes care provider to an adult diabetes care provider (7). The struggles of these transitions coupled with the relentless demands of day-to-day management of diabetes—diet, insulin, medication schedules, and monitoring blood glucose—may be stressful and burdensome and result in poor prognosis (8, 9).

A meta-analysis by Anderson et al. reported that the prevalence of mental illness among adults with diabetes is 2 times higher than its prevalence among adults without diabetes (10). Early-onset mental illness also increases the risk of significant mental health problems in the later years (11). Mental health comorbidities are also associated with an increased risk of suicide (12) among emerging adults with both T1D and T2D. The association between diabetes and depression, and their co-existence has been studied extensively by researchers among adults (10, 13). However, there are still limited data on the risk of mental health comorbidities among emerging adults with diabetes (3).

Considering the current gap in literature, the objective of this study was to expand the knowledge and understanding of mental health comorbidities and associated factors in this specific demographic of emerging adults with diabetes. This study aimed to identify disparities in past-year prevalence of major depressive episode (MDE) and suicidal ideation among emerging adults with diabetes based on key demographic and socioeconomic determinants using a nationally representative dataset.

## Method

### Data source and study design

This study used a retrospective cross-sectional study design and data from the 2015 to 2019 National Survey on Drug Use and Health (NSDUH) public use data files. The NSDUH is an annual cross-sectional survey which is representative of the US population. Conducted by the Substance Abuse and Mental Health Services Administration (SAMHSA), the NSDUH is one of the primary sources of information about mental health disorders, and other health-related issues among members of the US, including civilian, noninstitutionalized US population aged 12 years and older. The survey collects data from noninstitutionalized residents of all 50 states and the District of Columbia (14).

NSDUH uses a stratified multistage area probability sampling to achieve national representativeness. Household survey interviews are conducted in-person using both computer-assisted personal interviews and audio computer-assisted self-interviewing. Privacy is maintained during the interview to increase the level of comfort and honesty in reporting confidential information about sensitive behaviors related to mental health. Further details about the NSDUH methodology and data structure are available at the SAMHSA website (15). For this analysis, the public-use data files for years 2015-2019 were merged to get stable national estimates. This study was deemed exempt from review by the University of Florida IRB. Study findings were reported by using the STROBE (Strengthening the Reporting of Observational Studies in Epidemiology) guidelines.

### Study sample

The sample was drawn from the merged file for years 2015-2019. Data for only emerging adults aged between 18-25 years old with a diabetes diagnosis (both type 1 and type 2) were included in the analysis. Diagnosis of diabetes was derived using the question, “Below is a list of health conditions that you may have had during your lifetime. Please read the list and type in the numbers of all the conditions that a doctor or other health care professional has ever told you that you had: Ever told had diabetes/sugar diabetes.” The responses were recorded as Yes/No. Participants with a history of any type of cancer were excluded from the analytic sample. The final study sample included 1,125 individuals of ages 18 to 25 years with a history of diabetes.

### Study variables

#### Past year major depressive episode

MDE was assessed in the NSDUH by an indicator based on the criteria in the Diagnostic and Statistical Manual of Mental Disorders (DSM-5), 5th edition (16). MDE was defined as “a period of at least 2 weeks when the respondent experienced a depressed mood or loss of interest or pleasure in daily activities, and other symptoms” (15). A respondent was classified as having MDE, if they reported experiencing at least 5 out of the 9 criteria used to define MDE, where at least one of the criteria is a depressed mood or loss of interest or pleasure in daily activities. Past year MDE was operationalized as dichotomous (Yes/No) variables in the analysis.

#### Suicidal ideation

Suicidal ideation was assessed with the question: “At any time in the past 12 months, up to and including today, did you seriously think about trying to kill yourself?” Based on their answers to the above question respondents were categorized into 2 groups representing suicidal ideation 1) Suicidal ideation, 2) No suicidal ideation.

#### Key sociodemographic variables

Self-reported race/ethnicity (non-Hispanic White, non-Hispanic Black, Hispanic, other), sex (male, female), sexual orientation (lesbian/gay/bisexual, heterosexual), family income (<$20,000, $20,000-$49,999, $50,000-$74,999, $75,000 or more), and education (less than high school, high school, some college) were included as key sociodemographic variables for assessing disparities.

#### Covariates

Additional demographic characteristics like marital status (married/unmarried), census region (large metro, small metro, non-metro), insurance (yes, no), current school/college enrolment (yes, no), general health status (fair/poor, good, very good/excellent) substance use and alcohol dependence (yes, no), disability status (yes, no), current dorm status (yes, no) and comorbid conditions (yes, no) were included as covariates in the analysis. We chose these covariates given their association with increased risk for suicide or mental health condition based on previous studies (17).

### Statistical analysis

For describing the study population, survey-design adjusted descriptive statistics were used. Wald chi-square tests were used to describe differences in the population characteristics by past year prevalence of MDE and suicidal ideation among individuals with a history of diabetes. Proportion of individuals with mental health comorbidities were estimated using survey weights to generate estimates that represented the US civilian noninstitutionalized population. To investigate disparities in mental health comorbidities both bivariate and multivariable logistic regression models were used with key sociodemographic variables as predictors for each outcome measure. All known confounders were included in the models as covariates. All analyses were conducted using SAS 9.4. (SAS Institute, Cary, NC), and statistical significance was determined at p<0.05.

## Results

### Descriptive statistics

The final study sample included 1,125 individuals (weighted sample of 2,620,937 US emerging adults aged 18-25 years old). The baseline characteristics of the study population are presented in **Table 1**. Out of 1,125 emerging adults with diabetes, majority were female (58%), non-Hispanic White (53%), with a college or higher degree (48%), family income between $20,000 to $49,999 (37%), unmarried (86%), identified as heterosexual (85%), covered by health insurance (85%), had excellent or very good general health (41%), no comorbidities (75%), currently not attending school (60%), not living in college dorm (98%), residing in a large metro area (51%), with no substance abuse disorder (86%), and without any disability (73%).

**Table 1:** Baseline characteristics of study population, emerging adults (18-25 years) with diabetes, 2015-2019 NSDUH.

Characteristics	Unweighted population	Weighted population	
	N = 1,125	n = 2,620,937	
	No.	Weighted % (95% CI) ^a^	P-value
Sex			
Male	427	41.7 (37.2-46.3)	0.001
Female	698	58.2 (53.6-62.7)	
Race/Ethnicity			
Non-Hispanic White	572	52.5 (47.4-57.6)	<.0001
Non-Hispanic Black	191	17.6 (15.4-19.7)	
Hispanic	242	22.1 (17.5-26.6)	
Other ^b^	120	7.69 (5.61-9.77)	
Education			
Less than high School	205	17.6 (15.1-20.0)	<.0001
High school graduate	425	34.5 (30.2-38.8)	
College or higher	495	47.8 (43.9-51.7)	
Family Income			
Less than $20,000	375	29.8 (26.2-33.4)	<.0001
$20,000 to $49,999	424	36.6 (32.8-40.3)	
$50,000 to $74,999	123	10.6 (8.45-12.8)	
$75,000 or More	203	22.8 (19.1-26.5)	
Marital Status			
Married	187	14.3 (11.5-17.1)	<.0001
Unmarried ^c^	908	85.6 (82.8-88.4)	
Sexual Identity			
Heterosexual	914	84.7 (82.1-87.2)	<.0001
LGB	191	15.2 (12.7-17.8)	
Health Insurance			
Yes	958	84.7 (81.9-87.5)	<.0001
No	167	15.2 (12.4-18.0)	
General Health			
Excellent/Very Good	436	41.2 (36.9-45.5)	<.0001
Good	422	35.6 (31.9-39.4)	
Fair/Poor	267	23.0 (20.1-25.9)	
Comorbidities			
No	820	74.8 (71.7-77.9)	<.0001
Yes	305	25.1 (22.0-28.2)	
Current School Enrolment			
No	545	59.9 (55.2-64.7)	<.0001
Yes	321	40.0 (35.2-44.7)	
Dorm Status	<.0001
No	1093	97.6 (96.4-98.8)	
Yes	32	2.34 (1.15-3.52)	
Census Region			
Large metro	434	50.9 (46.9-54.8)	<.0001
Small metro	439	33.9 (30.2-37.6)	
Non metro	252	15.1 (12.3-17.8)	
Substance Use Disorder ^d^			
No	960	86.3 (83.7-88.8)	<.0001
Yes	165	13.6 (11.1-16.2)	
Disability			
No	815	72.9 (69.8-76.0)	<.0001
Yes	310	27.0 (23.9-30.1)	

LGB, lesbian gay bisexual.

a Estimates were weighted to be nationally representative using recommended stratification, clustering, and weighting by Substance Abuse and Mental Health Services Administration.

b Other includes non-Hispanic Native American/Alaska Native, Native Hawaiians/Other Pacific Islander, non-Hispanic Asian, and more than one races.

c Includes widowed, divorced, or separated.

d Based on the criteria in the American Psychiatric Association, Diagnostic and Statistical Manual of Mental Disorders, 4th edition.

### Disparities related to past year MDE

Lower odds of having past year MDE were estimated for non-Hispanic Black (adjusted odds ratio [AOR], 0.42, 95% CI, 0.19-0.92, p=0.032) compared to their non-Hispanic White counterparts. Females were 3.02 times more likely to have past year MDE than males (AOR, 3.02, 95% CI, 1.45-6.27, p=0.004). The odds of having past year MDE were 1.96 times higher among individuals who identified as LGB (AOR, 1.96, 95% CI, 1.04-3.68, P=0.038) (**Table 2**).

**Table 2:** Unadjusted and adjusted analysis of disparities related to Past year MDE among emerging adults (18-25 years) with diabetes.

	Crude Odds Ratio	(95% CI)	P-value	Adjusted ^a^ Odds Ratio	(95% CI)	P-value
Race/Ethnicity						
Non-Hispanic White	Ref	–	–	Ref	–	–
Non-Hispanic Black	0.58	(0.29-1.14)	0.112	0.42	(0.19-0.92)	0.032
Hispanic	0.59	(0.29-1.20)	0.144	0.59	(0.25-1.33)	0.198
Other	1.21	(0.53-2.75)	0.642	0.81	(0.27-2.33)	0.687
Gender						
Male	Ref	–	–	Ref	–	–
Female	3.00	(1.84-4.86)	<.0001	3.02	(1.45-6.27)	0.004
Education						
Less than High School	Ref	–	–	Ref	–	–
High School Graduate	0.99	(0.53-1.83)	0.966	1.03	(0.45-2.35)	0.936
Some College	1.13	(0.52-2.41)	0.750	1.43	(0.60-3.39)	0.408
Family Income						
Less than $20,000	Ref	–	–	Ref	–	–
$20,000 to $49,999	1.25	(0.69-2.25)	0.450	1.48	(0.75-2.89)	0.248
$50,000 to $74,999	1.08	(0.44-2.62)	0.859	1.92	(0.56-6.48)	0.287
$75,000 or More	0.83	(0.41-1.66)	0.584	0.87	(0.36-2.04)	0.743
Sexual Identity						
Heterosexual	Ref	–	–	Ref	–	–
LGB	2.75	(1.62-4.66)	0.001	1.96	(1.04-3.68)	0.038

LGB, lesbian, gay, bisexual; Ref, reference.

a Adjusted model included sex, race/ethnicity, education, family income, marital status, sexual identity, health insurance, general health, comorbidities, current school enrolment, dorm status, census region, substance use disorder, and disability.

### Disparities related to past year suicidal ideation

There were no statistically significant disparities in suicidal ideation related to race/ethnicity, sex, education, and family income among the study population. Like for MDE, individuals who identified as LGB had significantly higher likelihood of suicidal ideation than their heterosexual counterparts (AOR, 2.47, 95% CI, 1.36-4.47, P=0.004), (**Table 3**).

**Table 3:** Unadjusted and adjusted analysis of disparities related to suicidal ideation among emerging adults (18-25 years) with diabetes.

	Crude Odds Ratio	(95% CI)	P-value	Adjusted ^a^ Odds Ratio	(95% CI)	P-value
Race/Ethnicity						
Non-Hispanic White	Ref	–	–	Ref	–	–
Non-Hispanic Black	0.60	(0.34-1.06)	0.082	0.63	(0.30-1.31)	0.214
Hispanic	0.57	(0.30-1.08)	0.085	0.76	(0.37-1.53)	0.441
Other	1.15	(0.54-2.39)	0.713	0.98	(0.47-2.01)	0.953
Gender						
Male	Ref	–	–	Ref	–	–
Female	1.35	(0.90-2.00)	0.137	1.66	(0.76-3.59)	0.194
Education						
Less than High School	Ref	–	–	Ref	–	–
High School Graduate	1.06	(0.51-2.18)	0.875	1.14	(0.49-2.64)	0.758
Some College	0.86	(0.46-1.58)	0.626	1.41	(0.66-2.99)	0.359
Family Income						
Less than $20,000	Ref	–	–	Ref	–	–
$20,000 to $49,999	1.21	(0.77-1.91)	0.399	1.41	(0.80-2.46)	0.227
$50,000 to $74,999	0.85	(0.40-1.77)	0.656	1.17	(0.46-2.94)	0.728
$75,000 or More	0.70	(0.38-1.28)	0.245	0.79	(0.41-1.52)	0.481
Sexual Identity						
Heterosexual	Ref	–	–	Ref	–	–
LGB	2.91	(1.80-4.70)	<.0001	2.47	(1.36-4.47)	0.004

LGB, lesbian, gay, bisexual; Ref, reference.

a Adjusted model included sex, race/ethnicity, education, family income, marital status, sexual identity, health insurance, general health, comorbidities, current school enrolment, dorm status, census region, substance use disorder, and disability.

## Discussion

The purpose of this research was to identify disparities in past-year MDE and suicidal ideation among emerging adults with diabetes based on key demographic and socioeconomic factors. Using a nationally representative sample of 1,125 emerging adults, who had a history of diabetes, we found significant disparities in MDE and suicidal outcomes based on race/ethnicity, gender and sexual orientation. Below we discuss these findings in more detail.

Females and participants who identified as LGB were more likely to have past-year MDE. These results are supported by a study that found individuals with diabetes and comorbid depression to be younger, female, and with poor physical health (18). Gender differences in the prevalence of MDE have long been recognized within the general population (18). Possible reasons could be social, biological or psychological in nature such as discrimination based on gender (19), differential biological response to stress (20), differences in exposure to adversities (21). Another explanation of these findings could be the under-reporting of the symptoms of depression. Research suggests that males tend to under-report symptoms and severity of depression (22). It has been argued that DSM-5 reflects symptoms of depression in females better than males (23).

It has been found that individuals who identify as LGB are more likely to suffer from poor mental health (24–26). Research suggests that mental health disparities found at younger ages in LGB adults could persist later in the life course (24). The literature recognizes the existence of stigma and minority stress at individual, interpersonal and structural levels, and multiple mechanisms or pathways have been suggested for how stigma and minority stress can cause depressive symptoms among sexual minority individuals (27).

This study also found that non-Hispanic Black population were less likely to experience past-year MDE compared to non-Hispanic White. This finding is consistent with a previous study (28). Researchers speculate that non-Hispanic Blacks benefit from their social ties and coping strategies. For example, Weaver et al. concluded that stressors affect mental health of non-Hispanic Blacks to a lesser extent compared to non-Hispanic Whites (29). However, these assumptions lack empirical support, and reasons for racial/ethnic differences in past-year MDE remain unclear (30). Future research may help explore the factors contributing to lower risk of mental health illnesses among non-Hispanic Black emerging adults with diabetes.

As for suicidal ideation, echoing the existing literature trends, our findings suggest that LGB emerging adults experience increased risk of suicidal ideation relative to their heterosexual counterparts (26). Youth from sexual minority groups are 4 times more likely to attempt suicide as compared to their heterosexual counterparts (31). A recent study done by Roberts et al. included a sample of 64 patients ages 13 to 21 years diagnosed with diabetes, they reported that 9% of the study participants endorsed suicidal ideation (32). Another study reported 9% suicidal ideation rate with 83.4% clinically elevated depressive symptoms among youth and emerging adults (10 to 24 years) with diabetes (33). Within this context, it’s imperative to consider the compounded challenges faced by younger (or emerging) adults at the intersection of sexual minority status and diabetes. This suggests that the intersectionality of being both a sexual minority and having diabetes might present distinct mental health vulnerabilities compared to their heterosexual counterparts or those without diabetes. Although reasons for this trend remain unclear, exposure to violence, victimization and higher risk of isolation might lead to heightened levels of hopelessness and increased risk of suicidal ideation (34). Integration of a mental health professional into the multidisciplinary diabetes care team is critical for effective management of comorbid mental health conditions in emerging adults with diabetes. American Diabetes Association (ADA) standards of care specify that, “People with diabetes can benefit from a coordinated multidisciplinary team that includes mental health professionals” (35). Yet, not all diabetes care programs integrate mental health services in their diabetes care, and only 25% to 50% of those with diabetes and comorbid depression get access to treatment (36, 37). Our findings suggest that diabetes care providers and other healthcare professionals, including diabetes educators, should consider screening emerging adults with diabetes for early signs and risk factors of depression and current or past suicidal ideation, particularly LGB females, as part of a comprehensive care plan. Risk-based assessment made at an early stage of life could prevent the development of severe clinical depression among vulnerable individuals later in life. Consistent, widespread interventions to thwart impulsive suicidal behaviors should be established even in the absence of current suicidal thoughts. An inclusive approach supported by empirical evidence should be taken for treatment of depression in LGB emerging adults with diabetes to lower their risk of suicidal behaviors.

Providers who cater to the emerging adult population report having inadequate training and understanding of the unique needs of sexual minorities (38). Trainings and education focused on providing inclusive and quality care to sexual minority emerging adults is crucial and will ultimately help in eliminating disparities and inequities related to mental and physical health disparities. Future studies should demonstrate empirical evidence that supports efficacy of interventions specifically tailored to the unique needs of emerging LGB population. In addition, policy makers should focus on support strategies like awareness, counseling, peer support programs, and destigmatizing efforts while planning public health policies.

This study uses survey data that are representative of diabetes and mental health outcomes in the younger population, including those not necessarily seeing an endocrinologist for diabetes care. However, there are some limitations to this study approach. First, substance use disorder is a known risk factor for MDE and suicidal ideation (39), and it is crucial to fully control for this reverse causation. To address this limitation, all models were adjusted for substance use disorder. Next, the survey uses diabetes as a broader diagnosis and does not ask the respondents about their specific diabetes type. This is a common limitation of studies that use national survey data and self-reported diagnosis of diabetes, and is also acknowledged as a limitation by CDC and the National Institute of Diabetes and Digestive and Kidney Diseases in their publications (40, 41). Another limitation is that individuals with diabetes and mental illnesses or depressive symptoms are at risk of reciprocal susceptibility and share a high degree of comorbidity (42). Accounting for this reciprocity was not feasible because of the cross-sectional nature of the data. Additionally, the data on most outcomes was based on self-report and are not objectively measured, thus it is difficult to establish the accuracy of the information provided, which may suffer from recall bias or social desirability bias. However, disease incidence and service utilization estimates of NSDUH have been validated against other national data sources, which increases the confidence in the study findings (43). Another constraint of this study is the lack of data pertaining to participants’ HbA1C levels and diabetes duration. Consequently, we were unable to incorporate these factors into our analysis of the diabetes-depression relationship. Lastly, there is a possibility of confounding by several unobserved factors that were not controlled for because of the cross-sectional nature of the study.

## Conclusion

Despite much effort towards reducing and eliminating disparities related to physical and mental health outcomes, this study provides evidence of prevalent mental health disparities among emerging adults with diabetes. Our findings showed that women and sexual minority emerging adults with a history of diabetes are particularly exposed to higher risk of MDE. Moreover, the results highlight the disadvantages faced by sexual minorities among this specific population in terms of suicidal ideation. This research holds important implications for diabetes care professionals and policy makers to make their practices more favorable for disadvantaged groups who experience disparities and inequities. Further policy efforts are needed to educate, train, and sensitize diabetes care providers towards these prevalent mental health disparities among emerging adults with diabetes and for integration of a mental health professional into the multidisciplinary diabetes care team. These endeavors could ultimately lower the risk of this vulnerable group against development of severe mental health illnesses during later stages in life.

## Data availability statement

Publicly available datasets were analyzed in this study. This data can be found here: National Survey on Drug Use and Health 2019 (NSDUH-2019-DS0001) | SAMHDA. https://www.datafiles.samhsa.gov/dataset/national-survey-drug-use-and-health-2019-nsduh-2019-ds0001. Accessed August 31, 2021.

## Author contributions

SY: Conceptualization, Data curation, Formal Analysis, Investigation, Methodology, Project administration, Writing – original draft, Writing – review & editing. Y-RH: Conceptualization, Supervision, Writing – review & editing. SW: Writing – review & editing. NM: Writing – review & editing. MH: Writing – review & editing. AW: Conceptualization, Funding acquisition, Supervision, Writing – review & editing.
